# Reviewer acknowledgement 2014

**DOI:** 10.1186/s13223-015-0070-4

**Published:** 2015-02-08

**Authors:** Richard Warrington

**Affiliations:** University of Manitoba, Manitoba, Canada

## Abstract

The editors of *Allergy, Asthma & Clinical Immunology* would like to thank all of our reviewers who have contributed to the journal in Volume 10 (2014).

**Rohan Ameratunga**

New Zealand

**Francesco Annunziato**

Italy

**Meghan Azad**

Canada

**Allan Becker**

Canada

**Stephen Betschel**

Canada

**Patrizia Bonadonna**

Italy

**Robert Boyle**

United Kingdom

**John Brannan**

Australia

**Arnon Broides**

Israel

**Helen Annaruth Brough**

United Kingdom

**Gianfranco Calogiuri**

Italy

**Marilia Cascalho**

United States of America

**Zave Chad**

Canada

**Edmond Chan**

Canada

**Tesfahun Chanie**

Ethiopia

**Anca Mirela Chiriac**

France

**Antonella Canferoni**

United States of America

**Don Cockcroft**

Canada

**Michele Columbo**

United States of America

**Robert Cowie**

Canada

**Linda Cox**

United States of America

**Timothy Craig**

United States of America

**Anahit Demirchyan**

Armenia

**Pascal Demoly**

France

**Gabriele Di Lorenzo**

Italy

**Tony Dubois**

Netherlands

**Audrey DunnGalvin**

Ireland

**Anne Ellis**

Canada

**Marion Espeli**

France

**Berrylin Ferguson**

United States of America

**Javier Fernandez**

Spain

**Kristian Filion**

Canada

**Charles Frankish**

Canada

**Anastasios Germenis**

Greece

**Romain Gherardi**

France

**Paul Greenberger**

United States of America

**Michelle Halbrich**

Canada

**Agnes Hamzaoui**

Tunisia

**Kyla Hildebrand**

Canada

**Tomomitsu Hirota**

Japan

**Friedrich Horak**

Bahamas

**Komei Ito**

Japan

**Rajashri Shuba Iyengar**

United States of America

**Robert Jacobs**

United States of America

**Judith S Jacobson**

United States of America

**Rhoda Kagan**

Canada

**Sandeep Kapur**

Canada

**Fotini Kavadas**

Canada

**Paul Kevin Keith**

Canada

**Harold Kim**

Canada

**Jiro Kitaura**

Japan

**Marek Kowalski**

Poland

**Rajesh Kumar**

United States of America

**Jerold Last**

United States of America

**Ian Lewkowich**

United States of America

**Per Lidman**

Canada

**Phil Lieberman**

United States of America

**Hilary Longhurst**

United Kingdom

**Scott Lorch**

United States of America

**Kristian Macdonald**

Canada

**Flemming Madsen**

Denmark

**Soheila Maleki**

United States of America

**David Martino**

Australia

**Paolo Matricardi**

Italy

**Osamu Matsuno**

Japan

**Marcus Maurer**

Germany

**Bruce Mazer**

Canada

**Christine McCusker**

Canada

**Treasure McGuire**

Australia

**Paul Michel Mertes**

France

**David William Moote**

Canada

**Ralph Mösges**

Germany

**Bing Ni**

China

**Anna Nopp**

Sweden

**Kimihiro Okubo**

Japan

**John Oppenheimer**

United States of America

**Cevdet Özdemir**

Turkey

**Carmine Pariante**

United Kingdom

**Jonathan Parsons**

United States of America

**Niraj Patel**

United States of America

**Jaclyn Quirt**

Canada

**Avner Reshef**

Israel

**Mercedes Rincon**

United States of America

**Rachel Robison**

United States of America

**Monica Ruiz-Garcia**

United Kingdom

**Takashi Saito**

Japan

**Robert Schellenberg**

Canada

**Ralph Shapiro**

United States of America

**Peter Small**

Canada

**Diana Stafforini**

United States of America

**Donald Stark**

Canada

**Ruey Su**

Canada

**Gordon Sussman**

Canada

**Susan Tarlo**

Canada

**Peter Vadas**

Canada

**Cynthia Visness**

United States of America

**Giovanna Vitaliti**

Italy

**Harissios Vliagoftis**

Canada

**Brandie Walker**

Canada

**Kong-Sang Wan**

Taiwan

**Meng-Ting Wang**

Taiwan

**Susan Waserman**

Canada

**Wade Watson**

Canada

**Katharine Woessner**

United States of America

